# Prevalence of Precursory Signs of Atypical Femoral Fractures in Patients Receiving Bone‐Modifying Agents for Bone Metastases: A Cross‐Sectional Study

**DOI:** 10.1002/jbm4.10749

**Published:** 2023-04-27

**Authors:** Takumi Kaku, Yoto Oh, Shingo Sato, Hirotaka Koyanagi, Yuki Funauchi, Takashi Hirai, Masato Yuasa, Yu Matsukura, Toshitaka Yoshii, Tsuyoshi Nakagawa, Satoshi Miyake, Atsushi Okawa

**Affiliations:** ^1^ Department of Orthopaedic and Spinal Surgery Graduate School of Medical and Dental Sciences, Tokyo Medical and Dental University Tokyo Japan; ^2^ Department of Orthopaedic and Trauma Research Graduate School of Medical and Dental Sciences, Tokyo Medical and Dental University Tokyo Japan; ^3^ Center for Innovative Cancer Treatment, Tokyo Medical and Dental University Medical Hospital Tokyo Japan; ^4^ Department of Breast Surgery Tokyo Medical and Dental University Medical Hospital Tokyo Japan; ^5^ Department of Clinical Oncology Graduate School of Medical and Dental Sciences, Tokyo Medical and Dental University Tokyo Japan

**Keywords:** FRACTURE RISK ASSESSMENT, SCREENING, ANTIRESORPTIVES, TUMOR‐INDUCED BONE DISEASE, ATYPICAL FEMORAL FRACTURES

## Abstract

Patients on bone‐modifying agents (BMAs) for bone metastases are at risk of atypical femoral fractures (AFFs), which can lead to a sudden deterioration in performance status. In this study, we sought to determine the prevalence of radiographic precursory signs of AFF in patients on oncologic BMAs. Forty‐two patients (23 men, 19 women; mean age 68.8 ± 10.0 years) on oncologic BMAs (zoledronate for >3 years and/or denosumab for >1 year) and without clinical symptoms were enrolled between 2019 and 2021. All patients were receiving denosumab at enrollment and 5 had previously used zoledronate. The mean duration of BMA use was 31.2 ± 18.5 months. Radiographs of both femurs were screened for precursory signs of AFF (e.g., thickening of the lateral cortex). The patients were divided into two groups according to thickening status and compared by duration of BMA use. They were also divided into three groups by duration of BMA use (12–23 months, *n* = 18; 24–59 months, *n* = 19; ≥60 months, *n* = 5), and the prevalence of apparent thickenings was examined. As a result, 18 patients (42.9%) showed minute local or diffuse thickening and 10 (23.8%) showed apparent local thickening. The duration of BMA use was significantly longer in patients with apparent thickening than in those without (47.3 ± 23.6 months [*n* = 10] versus 26.2 ± 13.5 months [*n* = 32]; *p* < 0.05). The prevalence of apparent thickening increased with increasing duration of BMA use (12–23 months, 5.6%; 24–59 months, 31.6%; ≥60 months, 60.0%). In conclusion, radiographic precursory signs of AFF are common in patients on oncologic BMAs. Radiographic screening for AFF could be relevant in patients who have been on long‐term oncologic BMAs, even if asymptomatic. © 2023 The Authors. *JBMR Plus* published by Wiley Periodicals LLC on behalf of American Society for Bone and Mineral Research.

## Graphical abstract

We created a grading system of radiographic precursory signs for AFF and assessed femurs of 42 patients on oncologic bone‐modifying agents (≥1 year) and without symptoms. About a quarter of the patients had apparent precursory signs of AFF (i.e., grade 2). This suggests that the subclinical prevalence of radiographic changes in AFF is markedly high in cancer patients.

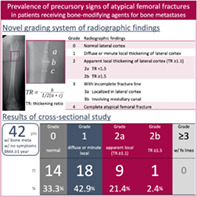

## Introduction

Cancer patients with bone metastases are generally treated with a bone‐modifying agent (BMA) such as zoledronate or denosumab.^(^ ^1^, ^2^, ^3^, ^4^, ^5^, ^6^, ^7^ ^)^ The recent improvement in the life expectancy of patients with cancer, including those with bone metastases,^(^ ^8^ ^)^ has resulted in more patients with bone metastases being on long‐term BMAs.^(^ ^1^, ^2^ ^)^ Long‐term use of BMAs has been associated with adverse events, in particular osteonecrosis of the jaw and atypical femoral fractures (AFFs).^(^ ^1^, ^2^ ^)^ Since the initial reports in about 2003,^(^ ^9^, ^10^, ^11^, ^12^ ^)^ osteonecrosis of the jaw has been considered the most important adverse event associated with long‐term oncologic use of BMAs.^(^ ^1^, ^2^ ^)^ However, AFF has also been widely recognized as an adverse effect of oncologic BMAs since around 2010, when the case definition of AFF was first published by the American Society for Bone and Mineral Research Task Force.^(^ ^13^ ^)^ Many case reports and case series of AFF due to the oncologic use of BMAs have been reported,^(^ ^14^, ^15^, ^16^, ^17^, ^18^, ^19^ ^)^ and AFF is now recognized as another important complication of long‐term BMA use.^(^ ^1^, ^2^ ^)^

According to the current case definition of AFF, which was revised in 2013, AFFs include fractures associated with minimal or no trauma, transverse or oblique fractures originating from the lateral cortex, noncomminuted or minimally comminuted fractures, and fractures with localized periosteal or endosteal thickening of the lateral cortex.^(^ ^20^ ^)^ A complete AFF and subsequent surgery can trigger a sudden decrease in performance status, performance of activities of daily living (ADL), and quality of life (QoL). Therefore, early detection of prodromal symptoms or signs of developing AFF and prophylactic treatment are important in patients with cancer.^(^ ^14^ ^)^ Prodromal symptoms of AFF, such as a dull or aching pain in the groin or thigh, are listed as minor features in the case definition but do not necessarily occur.^(^ ^20^ ^)^ Indeed, there have been several case reports of AFF that developed suddenly without any prodromal symptoms.^(^ ^14^, ^16^ ^)^ On the other hand, localized periosteal or endosteal thickening of the lateral cortex, known as beaking or flaring, is listed among the major features and is generally observed at the site of an AFF.^(^ ^20^ ^)^ These thickenings are assumed to develop asymptomatically before onset of a complete AFF or prodromal symptoms. Therefore, radiographic examination of the femur might reveal changes suggestive of developing AFF in patients at elevated risk. However, the incidence of asymptomatic radiographic changes in patients with cancer who are receiving a BMA, the time course of these findings, and the probability of progression to complete AFF are still unclear. The aim of this study was to determine the prevalence of radiographic precursory signs of AFF in cancer patients with bone metastasis.

## Material and Methods

### Study design, setting, ethics, and patient enrollment

This cross‐sectional study was performed at a high‐volume multidisciplinary university hospital that treats 2600 new cancer patients annually. The hospital also has a bone metastasis consultation system staffed by orthopedic surgeons that has managed over 900 patients with bone metastasis since it was established in April 2011. The study was approved by our institutional review board (M2019‐051), and informed consent was obtained from all study participants. All procedures involving human participants were performed in accordance with the ethical standards of the relevant institutional and/or national research committees and with the 1964 Helsinki Declaration and its later amendments or comparable ethical standards. The data were fully anonymized, thereby protecting the patients' privacy and dignity.

**Fig. 1:**
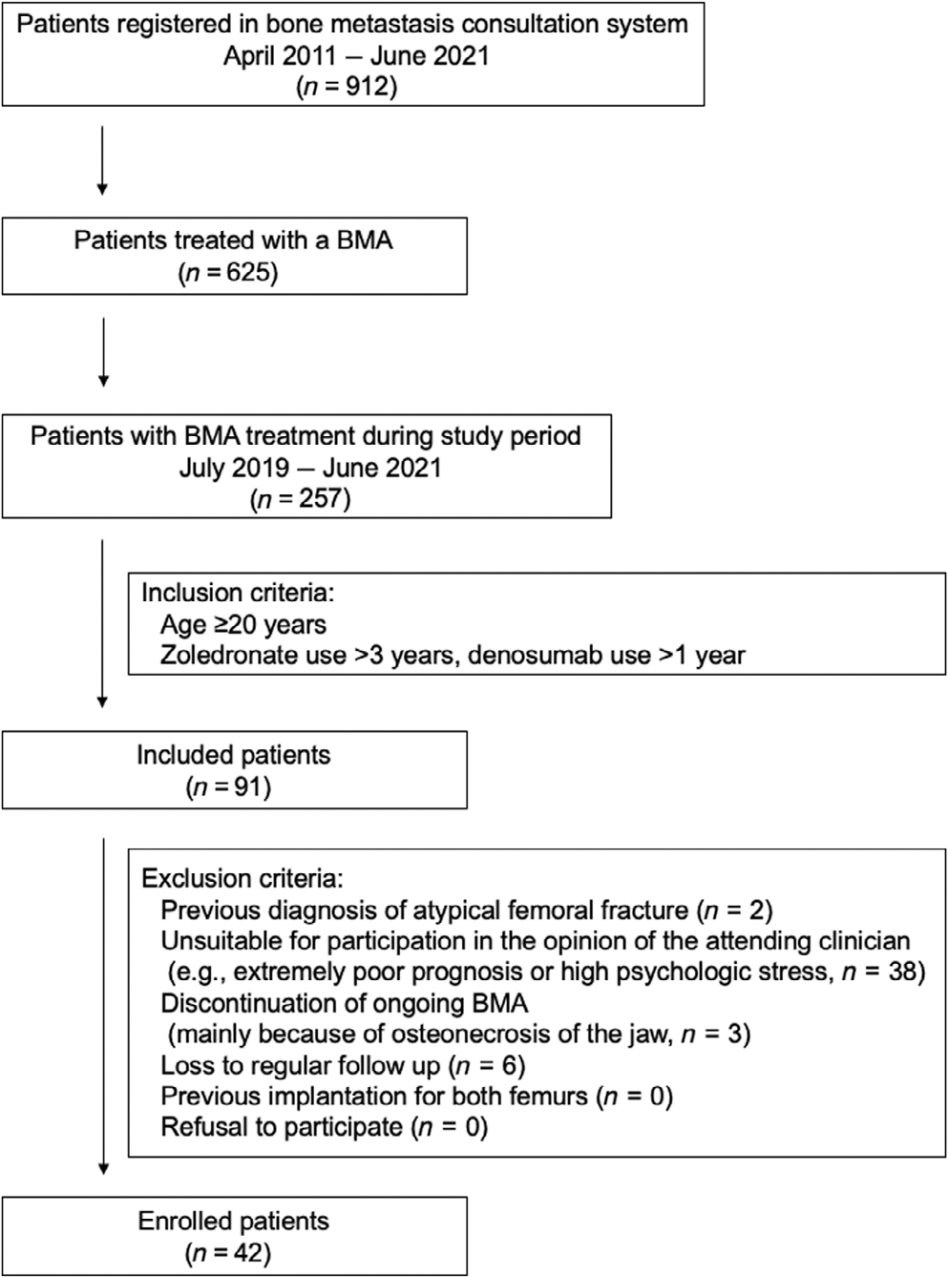
Flow chart showing patient recruitment process. BMA, bone‐modifying agent (zoledronate or denosumab).

From April 2011 to June 2021, a total of 912 patients were registered in the bone metastasis consultation system, 257 of whom were on oncologic BMAs between July 2019 and June 2021 according to institutional pharmacy records (Fig 1). Of these 257 patients, 91 who were receiving ongoing/prolonged treatment with a BMA during the study period were assessed for eligibility. The study inclusion criteria were age 20 years or older and treatment with an oncologic BMA for more than a certain duration (zoledronate, >3 years; denosumab, >1 year). After exclusion of 49 patients who did not meet the eligibility criteria, we finally enrolled 79 femurs (42 patients, 23 male, 19 female) without any prodromal symptoms suggestive of AFF, such as groin or thigh pain.

### Patient demographics

Mean age at enrollment was 68.8 ± 10.0 years (Table 1). Lung cancer was the most common primary lesion (*n* = 10, 23.8%), followed by breast cancer (*n* = 9, 21.4%), prostate cancer (*n* = 8, 19.0%), and neuroendocrine tumors (*n* = 5, 11.9%). Five patients had a history of switching from zoledronate to denosumab. All of the study participants were receiving ongoing treatment with denosumab during the study period. The mean duration of denosumab use was 28.7 ± 16.8 months, and the mean total duration of BMA use (zoledronate or denosumab) was 31.2 ± 18.5 months.

**Table 1:** Patient demographics

Total, *n*	42
Sex, *n* (%)	
Male	23 (54.8)
Female	19 (45.2)
Age	
Mean, years ± SD	68.8 ± 10.0
Primary malignancy, *n* (%)	
Lung cancer	10 (23.8)
Breast cancer	9 (21.4)
Prostate cancer	8 (19.0)
Neuroendocrine tumor	5 (11.9)
Renal cell carcinoma	3 (7.1)
Paraganglioma	2 (4.8)
Other	5 (11.9)
Total BMA use, *n* (%)	42 (100)
Mean duration, months ± SD	31.2 ± 18.5
Zoledronate use, *n* (%)	5 (11.9)
Mean duration, months ± SD	20.8 ± 37.4
Denosumab use, *n* (%)	42 (100)
Mean duration, months ± SD	28.7 ± 16.8

Abbreviation: BMA = bone‐modifying agent; SD = standard deviation.

### Radiographic examination and image interpretation

**Fig. 2:**
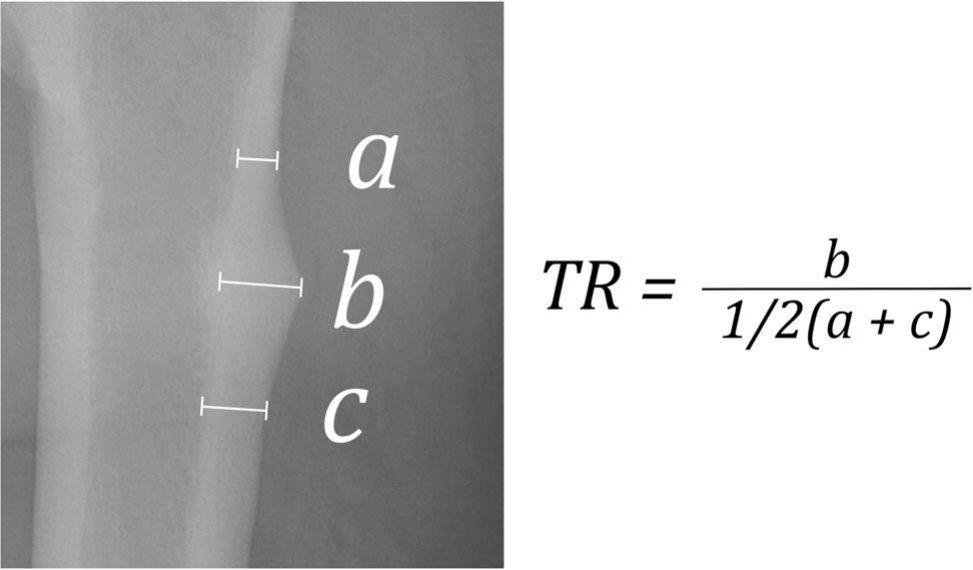
Definition of thickening ratio. We measured thicknesses at the proximal end (*a*), peak (*b*), and distal end (*c*) of a thickened area and calculated the TR in accordance with the formula. Points *a* and *c* mark the inflection point of the lateral cortex. TR, thickening ratio.

Full‐length radiographs of the femurs were obtained for all patients and searched from the proximal end to distal end of the diaphysis to identify whether there were any precursory signs of AFF, such as thickening of the lateral cortex or a fracture line. The imaging protocol was standardized as seven images, including both hips (anteroposterior and Lauenstein views) and both femurs (anteroposterior and lateral views), with anteroposterior images obtained with the patella in the true forward position. All images were evaluated by two orthopedic trauma surgeons jointly (TK, YO) familiar with the treatment of AFF. The thickening ratio (TR) for a focally thickened lateral cortex was calculated and graded (Table 2). The TR is the ratio of the peak cortical thickness to the average thickness between the proximal and distal ends of the thickened area (Fig 2). We assessed diffuse thickening and minute local thickening of the lateral cortex as grade 1, thickening with a TR ≥1.1 as apparent thickening (grade 2a, TR <1.5; grade 2b, TR ≥1.5), thickening with an incomplete fracture line as grade 3, and thickening with a complete AFF as grade 4. In the grading system, we put a dividing line between grades 1 and 2 at a 10% change in cortical thickening (TR <1.1 or ≥1.1). Based on the commonly accepted idea in experimental science that a value visually read to a tenth of the minimum scale is a significant figure, we assumed that a 10% difference in the thickness of the cortex was perceptible and obvious.^(^ ^21^ ^)^

**Table 2:** Grading system for precursory signs of atypical femoral fracture

Grade	Radiographic findings
0	Normal lateral cortex
1	Diffuse or minute local thickening of lateral cortex
2	Apparent local thickening of lateral cortex (TR ≥1.1, “beaking” or “flaring”)
	2a TR < 1.5
	2b TR ≥ 1.5
3	With incomplete fracture line
	3a Localized in lateral cortex
	3b Involving medullary canal
4	Complete atypical femoral fracture

Abbreviation: TR = thickening ratio.

### Analysis of association between progression of AFF and duration of BMA use

First, we divided the 42 patients into two groups (grade 0 or 1 and grade ≥2) and compared their duration of BMA use. We then divided the patients according to duration of BMA use (12–23 months, *n* = 18; 24–59 months, *n* = 19; and ≥60 months, *n* = 5) and calculated the proportion of patients with grade ≥2 thickening in each group.

### Statistical analysis

Continuous data are shown as the mean ± standard deviation and categorical data as the number (percentage). Variables were compared between the groups using the Mann–Whitney *U* test. All statistical analyses were performed using JMP version 12 (SAS Institute Inc., Cary, NC, USA). A *p*‐value <0.05 was considered statistically significant.

## Results

### Prevalence of radiographic findings

**Fig. 3:**
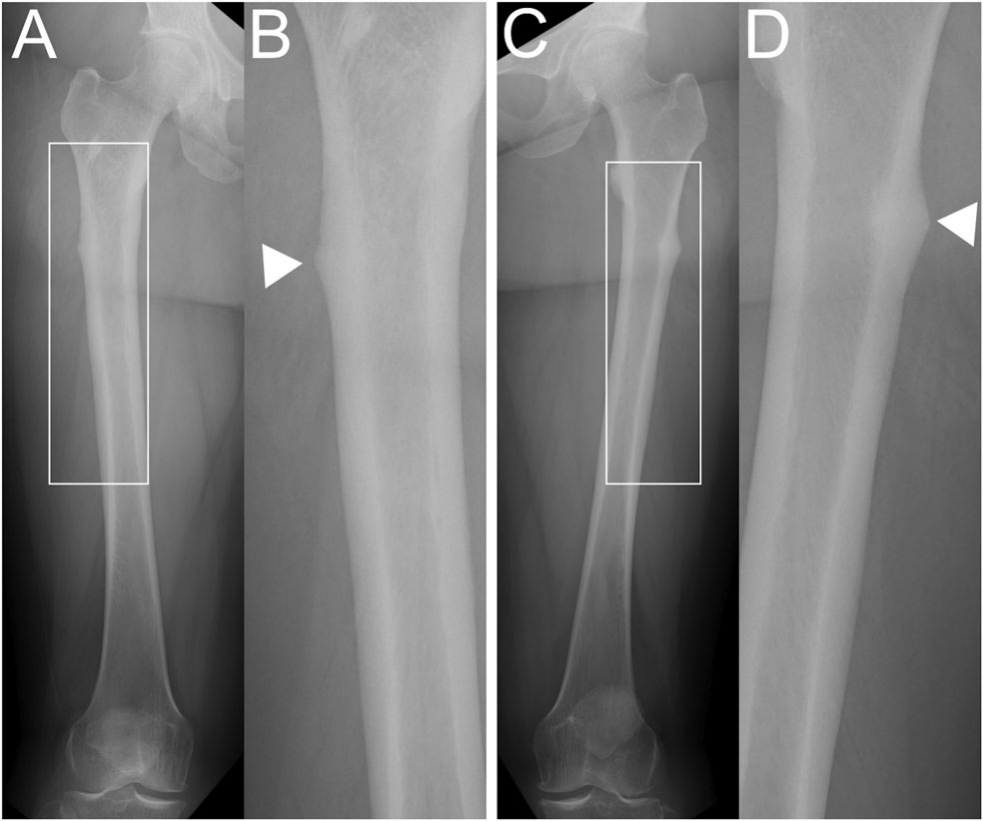
Images for patient with grade 2b radiographic findings. The patient was a 52‐year‐old woman with bone metastasis from breast cancer who had received oncologic denosumab for 74 months. (A) Anteroposterior image of right femur. (B) Enlarged image of white box in Fig 3A showing apparent local thickening of subtrochanteric lateral cortex (arrowhead, TR 1.5). The thickening had no radiolucent fracture line. (C) Anteroposterior image of left femur. (D) Enlarged image of white box in Fig 3C showing apparent local thickening of subtrochanteric lateral cortex (arrowhead, TR 1.9). The thickening had no radiolucent fracture line. Patient was assessed as grade 2b. TR, thickening ratio.

**Fig. 4:**
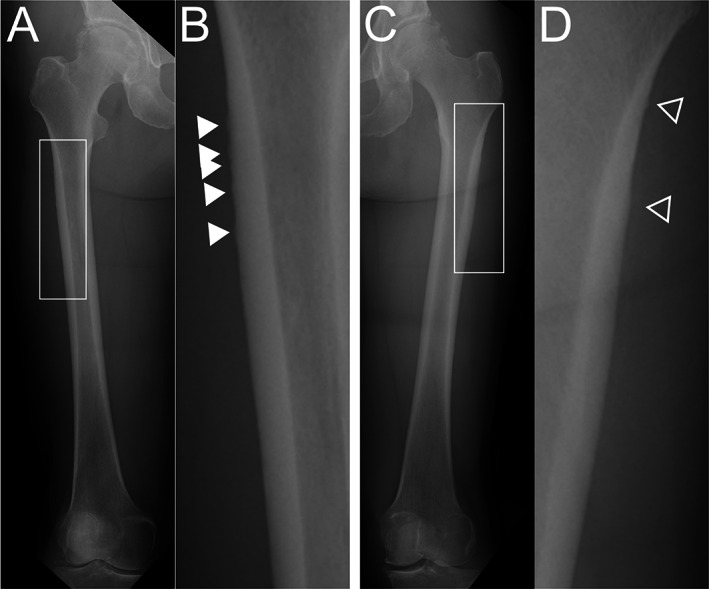
Images for patient with grade 2a radiographic findings. The patient was an 83‐year‐old woman with bone metastasis from lung cancer who had received oncologic denosumab for 65 months. (A) Anteroposterior image of right femur. (B) Enlarged image of white box in Fig 4A showing multiple apparent local thickenings of subtrochanteric lateral cortex (arrowheads, maximum TR 1.2). The multiple thickenings indicated a serrated cortex. (C) Anteroposterior image of left femur. (D) Enlarged image of white box in Fig 4C also shows two apparent local thickenings of subtrochanteric lateral cortex (arrowheads, maximum TR 1.1). She was assessed as grade 2a. TR, thickening ratio.

**Fig. 5:**
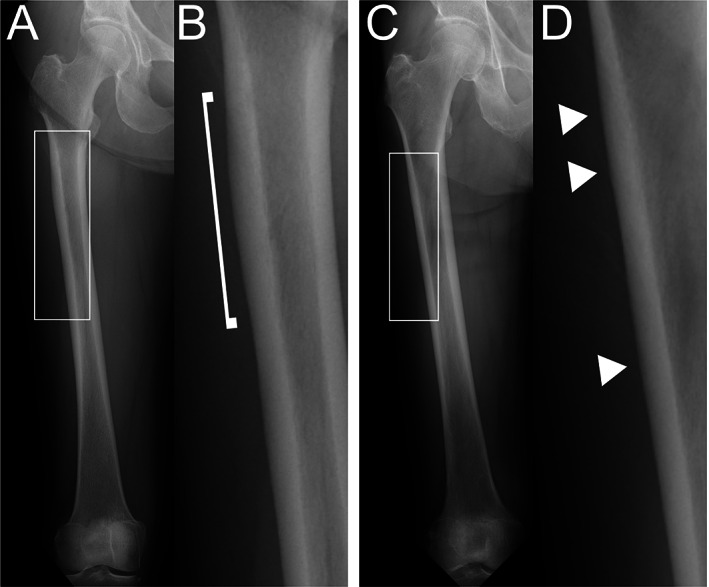
Images for two patients with grade 1 radiographic findings. (A, B) Images for first patient, a 72‐year‐old woman with bone metastasis from breast cancer who had received oncologic denosumab for 13 months. (A) Anteroposterior image of right femur. (B) Enlarged image of white box in Fig 5A shows diffuse thickening of subtrochanteric lateral cortex in right femur (open square bracket). She was assessed as grade 1. (C, D) Images for second patient, a 77‐year‐old man with bone metastasis from hepatocellular carcinoma who had received oncologic denosumab for 40 months. (C) Anteroposterior image of right femur. (D) Enlarged image of white box in Fig 5C shows minute local thickenings of subtrochanteric and midshaft lateral cortex in right femur (arrowheads, maximum TR <1.1). This patient was also assessed as grade 1. TR, thickening ratio.

Twenty‐eight of the 42 patients (66.7%) showed some type of radiographic change in the femoral diaphysis suggestive of developing AFF (Table 3). Twenty‐five of these 28 patients (89.3%) had changes in the subtrochanteric region and 18 (64.3%) had changes in the midshaft region; 15 patients (53.4%) had multiple changes in the subtrochanteric and midshaft regions. Apparent local thickening of the lateral cortex (grade 2, TR ≥1.1) was identified in 10 patients (23.8%; see Supplemental Table S1). Marked local thickening of the lateral cortex (grade 2b, TR ≥1.5) was identified in one patient (2.4%). Minute local or diffuse thickening (grade 1) was identified in 18 patients (42.9%). No grade 3 fracture lines were identified during the study period. Representative cases for each group are shown in Figs 3, 4, 5.

**Table 3:** Numbers of patients and their clinical characteristics according to grade of radiographic findings

Grade	0	1	2a	2b	3
*n* (%)	14 (33.3)	18 (42.9)	9 (21.4)	1 (2.4)	0 (0)
Male sex					
*n* (%)	10 (71.4)	9 (50.0)	4 (44.4)	0 (0)	
Patient age, years					
Mean ± SD	70.4 ± 10.0	68.9 ± 10.3	68.1 ± 9.2	52	
Primary malignancy					
Lung cancer	2	6	2	0	
Breast cancer	1	5	2	1	
Prostate cancer	4	2	2	0	
Neuroendocrine tumor	3	1	1	0	
Renal cell carcinoma	2	1	0	0	
Paraganglioma	2	0	0	0	
Other	0	3	2	0	
Duration of BMA use, months					
Mean ± SD	23.3 ± 12.0	28.4 ± 14.4	44.3 ± 23.0	74	
Minimum–maximum	13–52	13–62	14–89	74	

Abbreviation: BMA = bone‐modifying agent; SD = standard deviation.

### Association between progression of AFF and duration of BMA use

The mean duration of BMA use was significantly longer in the 10 patients with grade ≥2 radiographic signs than in the 32 patients with grade 0 or 1 radiographic signs (47.3 ± 23.6 months versus 26.2 ± 13.5 months; *p* = 0.0062). The frequency of grade ≥2 radiographic signs was 5.6% in the group that had used a BMA for 12–23 months (*n* = 1/18), 31.6% in the group that had used a BMA for 24–59 months (*n* = 6/19), and 60.0% in the group that had used a BMA for ≥60 months (*n* = 3/5).

## Discussion

Our findings suggest that the subclinical prevalence of radiographic changes and potential risk of AFF is markedly high in cancer patients with bone metastases receiving BMAs. About a quarter (23.8%) of the patients in this cross‐sectional study had apparent radiographic precursory signs of AFF (i.e., grade 2), despite being asymptomatic. Previous reports suggested that the incidence of AFF was only 1% to 2% in patients with cancer receiving BMAs.^(^ ^15^, ^16^, ^18^ ^)^ However, most of the relevant studies were retrospective and focused on patients with clinical or critical AFF who were treated surgically. A retrospective imaging study by Yang et al. identified three cases of subclinical asymptomatic AFF in 66 patients with bone metastases, giving an incidence of 4.5%.^(^ ^16^ ^)^ However, they were only able to review scout computed tomography, bone scintigraphy, or magnetic resonance images that were retrospectively available, so they could not comprehensively screen for radiographic signs of developing AFF. In contrast, the findings of our study, which included detailed screening for AFF using prospectively obtained radiographic images, suggests that the true prevalence of radiographic signs of AFF in cancer patients with bone metastasis who are receiving oncologic BMAs is markedly higher than previously reported.

We also found that longer‐term use of BMAs was associated with precursory signs and development of AFF. Our patients with grade 2 findings (apparent local thickening of the lateral cortex) had a longer history of BMA use than those with grade 0 or 1 finding. Several previous reports suggested a relationship between oncologic AFF and longer‐term BMA use.^(^ ^14^, ^15^, ^16^ ^)^ Takahashi et al. found that more than half of 12 patients with AFF associated with oncologic use of denosumab (seven from their own cohort and seven from previous reports extracted by systematic review) had used denosumab or another bisphosphonate for longer than 42 months.^(^ ^15^ ^)^ Although there are multiple risk factors for AFF, including drugs other than BMAs, certain diseases, bone morphology, loading stress, and bone fragility,^(^ ^20^, ^22^, ^23^, ^24^, ^25^, ^26^, ^27^, ^28^, ^29^, ^30^, ^31^, ^32^ ^)^ long‐term BMA use is widely considered to be the main cause.^(^ ^1^, ^2^, ^17^, ^22^, ^23^, ^33^, ^34^, ^35^, ^36^ ^)^ However, it has been unclear when we should check for precursory signs of AFF in patients on oncologic BMAs. In our study, warning signs were identified in one of 18 patients who had used a BMA for a short period (13–23 months). Therefore, we suggest that patients who have been taking an oncologic BMA, in particular denosumab, for more than 1 year should undergo regular radiographic screening.

Patients with lung cancer are traditionally considered least likely to develop oncologic AFFs because of their relatively short life expectancy^(^ ^14^ ^)^; however, this may not be the case in the future. In the context of oncologic BMA use, most of the previously reported cases of AFF were in patients with breast cancer, followed by prostate and other cancers.^(^ ^15^, ^16^, ^18^, ^19^ ^)^ In our present study, radiographic findings were grade ≥2 in 33.3% of patients (3/9) with breast cancer, 25.0% (2/8) in those with prostate cancer, and 20.0% (2/10) in those with lung cancer. Thus, the prevalence of radiographic precursory signs of developing AFF in patients with lung cancer was not much lower than that in patients with breast or prostate cancer. Therefore, oncologic AFFs are likely to become more common in patients with lung cancer as their life expectancy increases.

Although most AFFs associated with the use of a BMA for osteoporosis occur in women,^(^ ^20^ ^)^ oncologic AFFs can also be common in men. In our study, four of 10 patients with grade ≥2 findings and nine of 18 with grade 1 findings were men. There have been previous reports on oncologic AFFs occurring in men.^(^ ^14^, ^19^ ^)^ While there are undoubtedly sex‐related differences in the incidence of background diseases (e.g., osteoporosis, osteopenia, or metastatic bone disease), it appears that both sexes are at risk of AFF when on long‐term oncologic BMAs. However, further research is needed to determine whether there is a sex‐related difference in the risk of oncologic AFFs.

Strategies that can prevent oncologic AFFs are essential to preserve performance status, performance of ADL, and QoL in patients with bone metastases. The first step is to identify precursory signs of AFF by radiographic screening. It is important to use full‐length images of the femur because oncologic AFFs can occur not only in the subtrochanteric region but also in the midshaft, unlike the AFFs associated with the use of BMAs for osteoporosis, which usually occur in the subtrochanteric region.^(^ ^37^ ^)^ The second step may be regular radiographic follow‐up for warning signs at short intervals of three to six months. Patients should be counseled about the importance of monitoring these signs to prevent a complete AFF. The final step may be prophylactic fixation for AFF at the incomplete stage. Patients who develop an incomplete AFF as a result of taking a BMA for osteoporosis can be easily switched to parathyroid hormone therapy with the addition of other nonoperative strategies.^(^ ^20^, ^38^, ^39^ ^)^ However, in the context of incomplete AFF due to oncologic BMA use, interruption of the BMA therapy is often inappropriate because of the increased risk of skeletal‐related events.^(^ ^3^, ^4^, ^5^, ^6^, ^7^ ^)^ Furthermore, termination of denosumab without switching to parathyroid hormone therapy is associated with a rebound effect (e.g., a sharp decline in bone mass and increased risk of fragility fractures).^(^ ^40^, ^41^ ^)^ Therefore, nonoperative strategies are not recommended for incomplete AFFs due to oncologic BMA use, and prophylactic fixation may be preferable in patients with increasing pain or a radiolucent fracture line.

To our knowledge, the proposed grading system is novel. There are no reports of a grading system similar to ours, which assesses precursory signs of AFFs and their subclinical risks. The term “localized thickening of the lateral cortex” has not been clearly defined, and methods for evaluating images to assess precursory signs of AFFs have not yet been standardized. This is why we devised a new grading system that includes both objective and quantitative methods (including the TR).

This study has several limitations. First, for various reasons, such as limited life expectancy and psychologic stress, not all patients who met our inclusion criteria could be enrolled in the study, which raises the possibility of selection bias. Second, the BMA regimen for bone metastasis was not standardized, and we could not assess all the doses administered. Although almost all patients received regular monthly denosumab (120 mg by subcutaneous injection) or zoledronate (4 mg by intravenous injection), we were unable to record all doses or dosing intervals and only reviewed the duration of BMA use. This was because we are a multidisciplinary university hospital that cooperates with a number of regional hospitals and clinics where some patients receive BMAs. It is still controversial whether the duration or total number of doses of a BMA is the more significant factor for the development of AFF. Third, we did not assess the intrarater/interrater reliability. In the measurement of cortical thickness, there is a possibility of measuring errors between measurers. The intrarater/interrater reliability and utility of this grading system needs further evaluation. Fourth, the study population was small. Therefore, future investigations of the risk factors for AFF in cancer patients and precursory signs of AFF should include larger numbers of patients. Fifth, the association between elevated TR and the possibility of complete AFF development remains unclear. In future work, we will follow up the precursory signs of AFFs and attempt to clarify which signs tend to develop into radiolucent lines or complete AFFs. Sixth, we did not set a control group or assess whether or not regular healthy populations also have localized thickening of the lateral cortex.

## Conclusions

The subclinical prevalence of radiographic changes and the potential risk of AFF in cancer patients who have used oncologic BMAs in the medium term are remarkably high. Full‐length radiographic screening of the femur could be appropriate for patients with bone metastasis and a long‐term history of oncologic BMA use (especially denosumab) to avoid abrupt decreases in performance status, ADL, and QoL due to the sudden onset of complete AFF.

## Author Contributions

**Takumi Kaku:** Conceptualization; data curation; formal analysis; funding acquisition; investigation; methodology; project administration; visualization; writing – original draft; writing – review and editing. **Yoto Oh:** Conceptualization; data curation; formal analysis; funding acquisition; investigation; methodology; project administration; validation; visualization; writing – original draft; writing – review and editing. **Shingo Sato:** Conceptualization; methodology; resources; writing – review and editing. **Hirotaka Koyanagi:** Data curation; writing – review and editing. **Yuki Funauchi:** Data curation; writing – review and editing. **Takashi Hirai:** Conceptualization; data curation; methodology; writing – review and editing. **Masato Yuasa:** Data curation. **Yu Matsukura:** Data curation. **Toshitaka Yoshii:** Conceptualization; methodology; writing – review and editing. **Tsuyoshi Nakagawa:** Data curation; resources. **Satoshi Miyake:** Resources; supervision. **Atsushi Okawa:** Conceptualization; funding acquisition; methodology; project administration; resources; supervision; writing – review and editing.

## Conflicts of Interest

The department to which the corresponding author (YO) belongs has received funding for operating costs from Saku Central Hospital of the Nagano Prefectural Federation of Agricultural Cooperatives for Health and Welfare, Suwa Central Hospital, Doujin Hospital, Medtronic Sofamor Danek Co., Ltd., Stryker Japan K.K., and HOYA Technosurgical Co., Ltd., and NuVasive Japan K.K. The other authors declare that they have no conflicts of interest.

## Funding Information

This work was funded by a grant from the Japan Orthopedics and Traumatology Research Foundation, Inc. (No. 468). The sponsor was not involved in the study design, collection, analysis and interpretation of data, drafting of the manuscript, and the decision to submit the manuscript for publication.

### Peer Review

The peer review history for this article is available at https://www.webofscience.com/api/gateway/wos/peer-review/10.1002/jbm4.10749.

## Supporting information


**Table S1.** Characteristics of patients with grade 2 findings

## Data Availability

The data that support the findings of this study are available from the corresponding author upon reasonable request.

## References

[1] Coleman R , Hadji P , Body JJ , et al. Bone health in cancer: ESMO clinical practice guidelines. Ann Oncol. 2020;31:1650–1663. 32801018 10.1016/j.annonc.2020.07.019

[2] Rachner TD , Coleman R , Hadji P , Hofbauer LC . Individualized bone‐protective Management in Long‐Term Cancer Survivors with Bone Metastases. J Bone Miner Res. 2021;36:1906–1913. 34131949 10.1002/jbmr.4391

[3] Rosen LS , Gordon D , Kaminski M , et al. Long‐term efficacy and safety of zoledronic acid compared with pamidronate disodium in the treatment of skeletal complications in patients with advanced multiple myeloma or breast carcinoma: a randomized, double‐blind, multicenter, comparative trial. Cancer. 2003;98:1735–1744. 14534891 10.1002/cncr.11701

[4] Kohno N , Aogi K , Minami H , et al. Zoledronic acid significantly reduces skeletal complications compared with placebo in Japanese women with bone metastases from breast cancer: a randomized, placebo‐controlled trial. J Clin Oncol. 2005;23:3314–3321. 15738536 10.1200/JCO.2005.05.116

[5] Martin M , Bell R , Bourgeois H , et al. Bone‐related complications and quality of life in advanced breast cancer: results from a randomized phase III trial of denosumab versus zoledronic acid. Clin Cancer Res. 2012;18:4841–4849. 22893628 10.1158/1078-0432.CCR-11-3310

[6] Fizazi K , Carducci M , Smith M , et al. Denosumab versus zoledronic acid for treatment of bone metastases in men with castration‐resistant prostate cancer: a randomised, double‐blind study. Lancet. 2011;377:813–822. 21353695 10.1016/S0140-6736(10)62344-6 PMC3090685

[7] Henry D , Vadhan‐Raj S , Hirsh V , et al. Delaying skeletal‐related events in a randomized phase 3 study of denosumab versus zoledronic acid in patients with advanced cancer: an analysis of data from patients with solid tumors. Support Care Cancer. 2014;22:679–687. 24162260 10.1007/s00520-013-2022-1

[8] Center for Cancer Control and Information Services: National Cancer Center, Cancer statistics in Japan; 2021. https://ganjoho.jp/public/qa_links/report/statistics/2021_en.html.

[9] Marx RE . Pamidronate (Aredia) and zoledronate (Zometa) induced avascular necrosis of the jaws: a growing epidemic. J Oral Maxillofac Surg. 2003;61:1115–1117. 12966493 10.1016/s0278-2391(03)00720-1

[10] Tarassoff P , Csermak K . Avascular necrosis of the jaws: risk factors in metastatic cancer patients. J Oral Maxillofac Surg. 2003;61:1238–1239. 10.1016/j.joms.2003.09.001 14586868

[11] Ruggiero SL , Mehrotra B , Rosenberg TJ , Engroff SL . Osteonecrosis of the jaws associated with the use of bisphosphonates: a review of 63 cases. J Oral Maxillofac Surg. 2004;62:527–534. 15122554 10.1016/j.joms.2004.02.004

[12] Bamias A , Kastritis E , Bamia C , et al. Osteonecrosis of the jaw in cancer after treatment with bisphosphonates: incidence and risk factors. J Clin Oncol. 2005;23:8580–8587. 16314620 10.1200/JCO.2005.02.8670

[13] Shane E , Burr D , Ebeling PR , et al. Atypical subtrochanteric and diaphyseal femoral fractures: report of a task force of the American Society for Bone and Mineral Research. J Bone Miner Res. 2010;25:2267–2294 (published correction appears in J Bone Miner Res 2011;26:1987). 20842676 10.1002/jbmr.253

[14] Kaku T , Oh Y , Sato S , et al. Incidence of atypical femoral fractures in the treatment of bone metastasis: an alert report. J Bone Oncol. 2020;23:100301. 32642421 10.1016/j.jbo.2020.100301 PMC7334371

[15] Takahashi M , Ozaki Y , Kizawa R , et al. Atypical femoral fracture in patients with bone metastasis receiving denosumab therapy: a retrospective study and systematic review. BMC Cancer. 2019;19:980. 31640606 10.1186/s12885-019-6236-6 PMC6805596

[16] Yang SP , Kim TW , Boland PJ , et al. Retrospective review of atypical femoral fracture in metastatic bone disease patients receiving denosumab therapy. Oncologist. 2017;22:438–444. 28275116 10.1634/theoncologist.2016-0192 PMC5388375

[17] Lockwood M , Banderudrappagari R , Suva LJ , Makhoul I . Atypical femoral fractures from bisphosphonate in cancer patients—review. J Bone Oncol. 2019;18:100259. 31497503 10.1016/j.jbo.2019.100259 PMC6722257

[18] Puhaindran ME , Farooki A , Steensma MR , Hameed M , Healey JH , Boland PJ . Atypical subtrochanteric femoral fractures in patients with skeletal malignant involvement treated with intravenous bisphosphonates. J Bone Joint Surg Am. 2011;93:1235–1242. 21776577 10.2106/JBJS.J.01199

[19] Edwards BJ , Sun M , West DP , et al. Incidence of atypical femur fractures in cancer patients: the MD Anderson Cancer Center experience. J Bone Miner Res. 2016;31:1569–1576. 26896384 10.1002/jbmr.2818

[20] Shane E , Burr D , Abrahamsen B , et al. Atypical subtrochanteric and diaphyseal femoral fractures: second report of a task force of the American Society for Bone and Mineral Research. J Bone Miner Res. 2014;29:1–23. 23712442 10.1002/jbmr.1998

[21] Nivaldo JT . 2.3 significant figures: Writing numbers to reflect precision. Introductory Chemistry. 6th Ed in SI Units. New York: Pearson Education Limited; 2018 pp 51–56.

[22] Odvina CV , Zerwekh JE , Rao DS , et al. Severely suppressed bone turnover: a potential complication of alendronate therapy. J Clin Endocrinol Metab. 2005;90:1294–1301. 15598694 10.1210/jc.2004-0952

[23] Neviaser AS , Lane JM , Lenart BA , Edobor‐Osula F , Lorich DG . Low‐energy femoral shaft fractures associated with alendronate use. J Orthop Trauma. 2008;22:346–350. 18448990 10.1097/BOT.0b013e318172841c

[24] Lo JC , Huang SY , Lee GA , et al. Clinical correlates of atypical femoral fracture. Bone. 2012;51:181–184 (published correction appears in Bone 2013;53:13). 22414379 10.1016/j.bone.2012.02.632

[25] Oh Y , Wakabayashi Y , Kurosa Y , Ishizuki M , Okawa A . Stress fracture of the bowed femoral shaft is another cause of atypical femoral fracture in elderly Japanese: a case series. J Orthop Sci. 2014;19:579–586. 24789301 10.1007/s00776-014-0572-9

[26] Hagen JE , Miller AN , Ott SM , et al. Association of atypical femoral fractures with bisphosphonate use by patients with varus hip geometry. J Bone Joint Surg Am. 2014;96:1905–1909. 25410509 10.2106/JBJS.N.00075

[27] Oh Y , Wakabayashi Y , Kurosa Y , Fujita K , Okawa A . Potential pathogenic mechanism for stress fractures of the bowed femoral shaft in the elderly: mechanical analysis by the CT‐based finite element method. Injury. 2014;45:1764–1771. 25225173 10.1016/j.injury.2014.08.037

[28] Oh Y , Fujita K , Wakabayashi Y , Kurosa Y , Okawa A . Location of atypical femoral fracture can be determined by tensile stress distribution influenced by femoral bowing and neck‐shaft angle: a CT‐based nonlinear finite element analysis model for the assessment of femoral shaft loading stress. Injury. 2017;48:2736–2743. 28982480 10.1016/j.injury.2017.09.023

[29] Martelli S , Pivonka P , Ebeling PR . Femoral shaft strains during daily activities: implications for atypical femoral fractures. Clin Biomech (Bristol, Avon). 2014;29:869–876. 25156184 10.1016/j.clinbiomech.2014.08.001

[30] Haider IT , Schneider P , Michalski A , Edwards WB . Influence of geometry on proximal femoral shaft strains: implications for atypical femoral fracture. Bone. 2018;110:295–303. 29482067 10.1016/j.bone.2018.02.015

[31] Tano A , Oh Y , Fukushima K , et al. Potential bone fragility of mid‐shaft atypical femoral fracture: biomechanical analysis by a CT‐based nonlinear finite element method. Injury. 2019;50:1876–1882. 31519437 10.1016/j.injury.2019.09.004

[32] Oh Y , Yamamoto K , Yoshii T , Kitagawa M , Okawa A . Current concept of stress fractures with an additional category of atypical fractures: a perspective review with representative images. Ther Adv Endocrinol Metab. 2021;12:20420188211049619. 34671453 10.1177/20420188211049619 PMC8521412

[33] Koh JH , Myong JP , Yoo J , et al. Predisposing factors associated with atypical femur fracture among postmenopausal Korean women receiving bisphosphonate therapy: 8 years' experience in a single center. Osteoporos Int. 2017;28:3251–3259. 28748389 10.1007/s00198-017-4169-y

[34] Khow KS , Yong TY . Atypical femoral fracture in a patient treated with denosumab. J Bone Miner Metab. 2015;33:355–358. 24996528 10.1007/s00774-014-0606-6

[35] Schilcher J , Aspenberg P . Atypical fracture of the femur in a patient using denosumab – a case report. Acta Orthop. 2014;85:6–7. 24460109 10.3109/17453674.2014.885355 PMC3940983

[36] Thompson RN , Armstrong CL , Heyburn G . Bilateral atypical femoral fractures in a patient prescribed denosumab—a case report. Bone. 2014;61:44–47. 24389366 10.1016/j.bone.2013.12.027

[37] Oh Y , Yamamoto K , Hashimoto J , et al. Biological activity is not suppressed in mid‐shaft stress fracture of the bowed femoral shaft unlike in “typical” atypical subtrochanteric femoral fracture: a proposed theory of atypical femoral fracture subtypes. Bone. 2020;137:115453. 32470545 10.1016/j.bone.2020.115453

[38] Pearce O , Edwards T , Al‐Hourani K , Kelly M , Riddick A . Evaluation and management of atypical femoral fractures: an update of current knowledge. Eur J Orthop Surg Traumatol. 2021;31:825–840. 33590316 10.1007/s00590-021-02896-3

[39] van de Laarschot DM , McKenna MJ , Abrahamsen B , et al. Medical management of patients after atypical femur fractures: a systematic review and recommendations from the European calcified tissue society. J Clin Endocrinol Metab. 2020;105:1682–1699. 31867670 10.1210/clinem/dgz295 PMC7121199

[40] Tsourdi E , Langdahl B , Cohen‐Solal M , et al. Discontinuation of denosumab therapy for osteoporosis: a systematic review and position statement by ECTS. Bone. 2017;105:11–17. 28789921 10.1016/j.bone.2017.08.003

[41] Cummings SR , Ferrari S , Eastell R , et al. Vertebral fractures after discontinuation of denosumab: a post hoc analysis of the randomized placebo‐controlled FREEDOM trial and its extension. J Bone Miner Res. 2018;33:190–198. 29105841 10.1002/jbmr.3337

