# Supplementary material for: Prevalence of Precursory Signs of Atypical Femoral Fractures in Patients Receiving Bone‐Modifying Agents for Bone Metastases: A Cross‐Sectional Study
Source: JBMR Plus. 2023 Apr 27;7(7):e10749. doi: 10.1002/jbm4.10749 (PMC10339079; doi:10.1002/jbm4.10749)
Supplement: Supplementary file 1 — Table S1. Characteristics of patients with grade 2 findings [file JBM4-7-e10749-s001.docx]

**Supplemental material**

Supplemental Table 1: Characteristics of patients with grade 2 findings

| Case | Age, years | Sex | Primary lesion | Zoledronate use (months) | Denosumab use (months) | Side | Grade | Max TR | Location |
| --- | --- | --- | --- | --- | --- | --- | --- | --- | --- |
| 1 | 52 | Female | Breast cancer | - | 74 | R | 2b | 1.5 | ST |
|  |  |  |  |  |  | L | 2b | 1.9 | ST |
| 2 | 52 | Male | Prostate cancer | - | 29 | R | 2a | 1.1 | ST, MS |
|  |  |  |  |  |  | L | 1 | Diffuse | ST, MS |
| 3 | 60 | Female | Lung cancer | - | 18 | R | 2a | 1.1 | ST, MS |
|  |  |  |  |  |  | L | 1 | Diffuse | ST, MS |
| 4 | 61 | Female | Breast cancer | - | 52 | R | 2a | 1.1 | ST, MS |
|  |  |  |  |  |  | L | 1 | Diffuse | MS |
| 5 | 69 | Male | Pancreatic cancer | - | 32 | R | 1 | < 1.1 | ST, MS |
|  |  |  |  |  |  | L | 2a | 1.1 | ST, MS |
| 6 | 71 | Male | Neuroendocrine tumor | - | 31 | R | 2a | 1.4 | ST |
|  |  |  |  |  |  | L | 1 | Diffuse | ST |
| 7* | 71 | Male | Prostate cancer | - | 57 | R | 2a | 1.1 | MS |
|  |  |  |  |  |  | L | N/A |  |  |
| 8 | 71 | Female | Thyroid cancer | - | 26 | R | 1 | < 1.1 | ST, MS |
|  |  |  |  |  |  | L | 2a | 1.2 | ST, MS |
| 9 | 75 | Female | Breast cancer | 87 | 2 | R | 2a | 1.3 | ST, MS |
|  |  |  |  |  |  | L | 2a | 1.1 | ST, MS |
| 10 | 83 | Female | Lung cancer | - | 65 | R | 2a | 1.2 | ST |
|  |  |  |  |  |  | L | 2a | 1.1 | ST, MS |

*The left femur in case 7 had been fixed by an intramedullary nail because of a pathological fracture. MS, mid-shaft; N/A, not applicable; TR, thickening ratio; ST, subtrochanteric
